# United Hospitals Conference of Great Britain and Ireland

**Published:** 1906-12-15

**Authors:** 


### HOSPITAL ADMINISTRATION.

### CONSTRUCTION AND ECONOMICS.

### UNITED HOSPITALS CONFERENCE OF GREAT BRITAIN AND IRELAND.

### THE ABUSE AND MISUSE OF VOLUNTARY HOSPITALS.

### AN ANNUAL CONFERENCE WITH A STANDING COMMITTEE CONSTITUTED.

A conference of some 200 representatives and delegates of hospitals in the United Kingdom was held on Thursday, December 6, at University College to discuss resolutions submitted for consideration by the Joint Hospitals Committee nominated at the Conference of March 1905 called by the British Medical Association to consider the problems of hospital abuse and misuse. A number of proposals had been formulated by the Committee and submitted to the boards of hospitals throughout the country, of whom 150 sent delegates to the Conference. Other hospitals undertook to give consideration to the subject, while 29 were content to take no action.

In the absence of Lord Cheylesmore, Sir William Church took the chair, and in opening the proceedings said he thought it might assist the Conference if he briefly recapitulated the steps which had led to that meeting. Some three years ago the British Medical Association took up seriously the subject which, during many years, had greatly interested not only the profession, but those connected with hospitals; the subject which—though he did not himself like the term—was generally spoken of as "hospital abuse." The first step taken was to appoint a Committee, which, after doing a great deal of work and getting together a mass of information, called a Conference in March 1905 of various representatives of the profession and of hospital committees to consider general proposals which had already been formulated by the Hospitals Committee of the British Medical Association. Gentlemen who had had experience in hospital administration were elected to the Hospitals Committee, which then became the Joint Hospitals Committee, and had called them together that day. He thought they were all agreed that the present condition of the great hospitals in the Metropolis and throughout the country was unsatisfactory. This condition seemed to him to fall into two large classes. On the one hand they had hospitals appealing to the public for money to maintain and extend their work, and on the other hand there was a general feeling among medical practitioners that these extensions led to the evil of hospital abuse. He could not say that that widespread feeling was not founded on very just causes. This feeling was especially acute in regard to the out-patient department of hospitals; he would pass over the condition which attached to the in-patients' department. The out-patient system, like so many things British, was of gradual growth, but it had now got beyond what was ever anticipated. The subject presented long ago the same difficulties that it presented to-day. The first reference that he knew of occurred in the records of St. Bartholomew's Hospital, where, in 1682, a burning question arose as to the increase of the expenses of the apothecary's shop. This increase was due to the growth of out-patients—in those days mainly those who had been discharged from the wards and had returned to be seen by the medical officers. The numbers in those days, however, were very different from what they were now; they totalled only 300 or 400 per annum. If they cared to look at the records of the hospital they would find that since 1682 the question had been a burning one. The difficulty, therefore, was not a new one, but it had become great by reason of the extreme complexity of the matter. They knew that hospitals were constantly appealing to the public for funds to meet not only their present needs, but also to enable them to extend their work. He must say that he could not help feeling that many of the advertisements they saw from hospitals were anything but dignified. (Hear, hear.) What was wanted was co-operation among hospitals themselves—(hear, hear)—not competition. There could be no doubt to anyone who had gone into the question—and a great deal had been done by the two great Funds with which he was connected in the way of getting information on the subject—that the great body of the profession did feel a grievance with regard to hospital relief. He thought a good deal of the difficulty had arisen from the great changes which had come over society in the last fifty or sixty years. The whole matter was very complex. One thing struck him very forcibly, and that was the difference between the relations of the family doctor and those that used to obtain, not only towards the heads of the family, but the dependents as well. Nowadays if the dependents were ill the first idea was to appeal to someone for a letter to send them to a hospital, and, more often than not, to a special hospital, when they could just as well be treated at a general hospital. He could not help feeling that there was growing up a craze for specialists and specialities. (Hear, hear.)

Sir Henry Burdett moved: "That this Conference of representatives and supporters of hospitals, medical practitioners, and others interested in hospital administration in Great Britain and Ireland, approves generally of the principles contained in the proposals drawn up by the Joint Hospitals Committee and circulated to hospitals in August 1906."

### Sources of the Figures Quoted.

Sir Henry Burdett said: I am deeply sensible of the honour of having been invited to move the first resolution at the United Kingdom Hospitals Conference to-day. I am imbued with the sense of responsibility attaching to everything which I may say in regard to the important business which this Conference has to consider in dealing with the question of the undoubted excess in free medical relief at present granted by the voluntary hospitals and medical institutions. It may be well to say at the outset that all the figures I shall quote are taken from the Registrar-General's returns and the reports and returns of the hospitals and institutions for each year dealt with. I may point out that the hospital figures are those upon which the hospital authorities have based their appeals to the public for support, and the responsibility for their accuracy rests therefore upon the hospital authorities. I am bound to say, from my knowledge of those who are responsible for the administration of these institutions at the present time, that I am deeply sensible of the conscientious care with which these figures are now often prepared and sifted by the responsible officials.

In dealing with the numbers of patients attending the medical institutions in a great city, it is probable that some patients have occurred more than once in the returns, having attended the same hospital for more than one illness, or more than one charity, in one year. Some may have been counted twice in the same institution, first as out-patients and afterwards as in-patients. These facts do not in my judgment appreciably affect the results shown, but it is right that they should be kept in mind.

### THIRTY YEARS AGO AND NOW.

I think it may be useful to recall the Conference on Metropolitan Medical Relief held on April 17, 1877, at which Sir Charles Trevelyan read an exhaustive paper which has become a classic. At that Conference, on the motion of Sir William Gull, it was unanimously resolved that the improvement of the people in London, in health and habits of thrift and independence, demands that while, on the one hand, out-patient departments should be regulated so as to secure the prompt treatment of cases requiring the special resources of a hospital, on the other, free dispensaries should be converted into provident dispensaries, and new provident dispensaries should be established in proportion to the wants of the population. It will be my purpose to show that, for practical purposes, the wise principles laid down in this resolution have remained practically a dead letter, for the volume of hospital free medical relief has gone on extending during the whole of the thirty years which have elapsed, at a rate out of all proportion to the just needs of those classes of the population alone, who, in the day of illness, are entitled to receive free medical treatment.

Fortunately, as I consider, the members of the medical profession, to whom we all owe so much, have now aroused themselves, and have combined by active organisation to enforce adequate reforms by insisting upon a reduction in the present excessive amount of free medical relief at the hospitals. As a writer in the *Quarterly Review* has well said: "It must not be forgotten that there is a limit to the liberality of the most liberal profession in the world. As now situated the hospital not only does the work which belongs to the parochial authorities, but usurps and intercepts much of that which rightly appertains to an expensively educated professional class." This article was published in 1876, and in the years that have passed the whole tendency of free medical relief has been, unfortunately, in the direction of immeasurably adding to the evils referred to. Can the public, can hospital managers, can any one wonder, that the greatest organisation which the medical profession has ever possessed, that of the British Medical Association, constituted upon a widely representative basis, should have united to drive home the undoubted truth, that to-day the limit of the liberality of the most liberal profession in the world has been passed, and that a prompt and effectual remedy must be applied to the existing evils attaching to excessive free medical relief by the hospitals? We owe the calling of this Conference to the action of the British Medical Association, and we owe it to the members of that great profession that every one interested in the hospitals—and who is not?—should bring their influence to bear as a matter of simple justice to introduce and enforce the necessary reforms.

### THE AMOUNT OF FREE HOSPITAL RELIEF AT THE PRESENT TIME.

*In London.*—What then is the actual position presented by the facts which reveal the amount of free hospital relief given in this country at the present time? Dealing with the patients treated by the ninety-four voluntary hospitals in the county of London in the years 1894 and 1903, the following facts disclose themselves: That the population of the administrative county of London has only increased 6 per cent. in the ten years, while the percentage of increase in the hospital population has been 23¼ per cent. This represents an increase of over 330,000 patients, or 25 per cent. more than the whole number by which the total population has grown during the ten years under review. The out-patients amounted to 93 per cent. of the total number of the patients treated by the voluntary hospitals in the county of London in 1903. When I first published these figures it was stated that they were of small value, because the London hospitals drew so many of their patients from Greater London, and, if that were included, the results would be much less striking than they are as they stand. When, however, I took out the figures for Greater London, an area of nearly 700 square miles in which there are six administrative county authorities—*i.e.* those of London, Middlesex, Surrey, Kent, Essex, and Hertford, the evidence of excessive free relief by the London hospitals increases in force. The population of the whole area of Greater London (*i.e.* the administrative county and the outer ring) has increased in the ten years ending 1903 from 5,964,443 to 6,806,296, an increase of 14 per cent. in the decade; whilst the patients treated at all the hospitals within the whole area have increased by 25½ per cent. These figures show that the voluntary hospitals in Greater London have actually undertaken 81 per cent. more work than the increase in the population of the district warranted.

Figures are, I know, troublesome, and I will therefore try to bring out the facts in a very simple form, as they relate not only to London but to the principal cities in the United Kingdom. I wish it to be understood that I am prepared to show any one who cares to see them the details which make up the whole of the totals and the working of the calculations which I am now about to give. I mention this, because I wish it to be clear, that, there can be no question as to the official character and general accuracy of the facts, I here give, so far as they are ascertainable.

When Sir Charles Trevelyan read his paper thirty years ago it was stated on authority that one in four of the population of London received free medical relief; in 1894 one in 2½ of the population of the county of London received free medical relief, in 1903 the amount of such relief had increased to one in 2.2, and in 1904 to practically one in 2. It is interesting too to note that the amount of free medical relief granted to the inhabitants of Greater London increased in the decade ending 1903 to almost exactly the same extent that it did in the county of London where most of the greater hospitals are situated.

### Free Hospital Relief in the United Kingdom.

Dealing next with the United Kingdom, it may be interesting to point out, that, whereas there are nearly seven free hospital beds per 1,000 inhabitants in Dublin, there are only 2.7 hospital beds per thousand in London, 3.7 beds per thousand in Edinburgh, 2 beds per thousand in Glasgow, 1.8 beds per thousand in Newcastle-on-Tyne, 1.6 beds in Manchester, 1.5 beds in Liverpool, and 1 free hospital bed per thousand in Birmingham. Dealing next with the proportion of free patients to population in the 12 large towns in the United Kingdom, it appears that in 1903 in Portsmouth only one out of every 14 people received free medical relief; in Cardiff one out of every 7.1 people; in Glasgow one out of 5.3; in Manchester one in 3.5; in Liverpool one in 3.4; in Birmingham one in 3.2; in Brighton one in 3.1; in Bristol one in 2.9; in Edinburgh one in 2.8; in London one in 2.2; in Newcastle one in 1.9; in Dublin one in 1.3 of the population. It is further remarkable to notice that whereas, in the decade ending 1903, there has been a large increase in free medical relief in Newcastle, in Dublin, in London, in Birmingham, in Manchester, and especially in Glasgow and Cardiff, there has been a decrease in Edinburgh and in Liverpool, whilst matters have remained stationary at Portsmouth. At Bristol, owing to the enlargement of the area, identical figures are not available. The figures which I have given prove to demonstration that the growth in and the numbers of patients admitted to free medical relief must be far in excess of what they ought to be in the interests of all classes.

### The Injustice of the Present Position.

Now, excessive free medical relief must be an injustice to (1) hospital committees, their officials and supporters; (2) the honorary visiting medical officers of the hospitals; (3) the general practitioners; and (4) the patients.

1. *Hospital Managers and Supporters.*—No hospital in my judgment is justified in asking the public to give it generous pecuniary support unless it can show that every penny entrusted to it is wisely and economically expended in promoting the objects for which it was established, and those objects alone. The public have nobly supported the 94 voluntary hospitals in the county of London, a fact proved by the knowledge that, the total amount of money contributed to these institutions in 1903 was nearly £525,000 more than in 1894, being an increase of something like 65 per cent. I have had exceptional opportunities of knowing the views of many of the most liberal supporters of the great hospitals, and I am confident, that, if steps are not taken to reduce the volume of free medical relief at present given, the contributions to the voluntary hospitals will most certainly tend to diminish rather than to increase. The managers and officials must therefore welcome a reduction in the amount of free medical relief now given at their institutions.

2. *The Honorary Visiting Medical Officers.*—The present excessive numbers at certain hospitals, and the trivial nature of many cases, are a cruel hardship to the visiting medical officers. As Sir William Ferguson pointed out long ago, a hospital staff consists of consultants, not general practitioners, and it is only consistent that their services should be asked for, chiefly, in cases of peculiar difficulty. An inordinate number of trivial cases wastes the time of the consultants, wearies the attention of the students, and fosters a habit of hasty diagnosis and careless observation, which tend to erroneous and ineffective treatment. In fact, especially where the numbers are excessive, out-patient work, as generally conducted, neither conduces to the sound advancement of professional knowledge, nor to the advantage either of the patients, the students, or the public.

3. *The General Practitioners.*—The view taken by the general practitioners on this question, and the supreme urgency with which they regard it, is testified by the Conference held here this afternoon. The general practitioners are injured directly and indirectly by the fourfold character of the abuses and misuses attaching to the present system of excessive free medical relief. The public have not grasped as yet that the abuse is fourfold. It exists, *first* of all, whenever a patient, who can afford to pay something for his treatment, receives it for nothing; *secondly*, when a patient for whom the poor-law provides adequate means of medical relief obtains such relief at a voluntary hospital; *thirdly* when a patient who has no direct claim whatever to receive free medical relief at a metropolitan hospital still obtains it there; *fourthly*, when tens of thousands of persons every year attend the casualty department of the medical charities, although the greater number of these suffer from trivial ailments, which people in other stations of life, who pay for their medical service, would probably not seek medical aid for at all.

*The Patients.*—Last of all there is the injustice to the patients. Nothing can make this clearer than the words of Sir William Gull:—"The poor have an idea that disease comes from Providence and that it must be cured by drugs. Now, if there is any idea that ought to be rooted out, it is this. Children are often brought to be drugged when in reality they require to be washed and fed. Disease should be prevented by attending to hygienic laws, by eating good food which has been properly cooked, by regulating the quantity and guaranteeing the quality of that which is taken. The existence of gin palaces at one corner of the street and free dispensaries at the other are evidences by contrast of the monstrous anomalies existing in our society." Every out-patient medical officer has realised the force and truth of the points here urged by Sir William Gull. Take one instance as typical of many classes of cases, that of the common out-patient case of a woman who is suffering from excessive tea-drinking and irregular meals. If the physician tells her to stop tea and gives no medicine she will not obey him, and the symptoms will grow worse. If, however, he gives her something in a bottle, with directions to take it every three hours and to abstain from taking tea two hours before and two hours after each dose, the patient knocks off the tea and gives herself some chance of restoration to health. It is no benefit to the patient, no benefit to the poor, to be afforded infinite facilities for taking physic, and it is a positive injury to their character and moral nature to provide these facilities without payment, under conditions which render adequate treatment impossible. What justification can be urged for granting hospital benefits to those already better provided for by the rates? I mean of course the many cases which need food rather than physic. We are justly proud of our voluntary hospitals, but of late it seems to me as if many people have forgotten, there are two kinds of charity: "One beneficent, the other injurious. One which raises its objects, develops their resources and trains them to habits of health by calling forth a spirit of independence; the other blind, foolish and injurious, the charity which generates and fosters that spirit of dependence which is the chief cause of pauperism in this country."

### THE PRINCIPLES ON WHICH THE PROPOSED REFORMS REST.

You are asked to-day by the resolution I have moved to approve generally of the principles contained in the proposals you have in detail on the printed paper. Those principles are broadly as follows: That the number of new cases shall be limited. That no one able to pay for adequate treatment, no one eligible for Poor-law medical relief, no one unless really ill, should receive free medical relief, except they be sent to a hospital for purposes of consultation. That steps shall be taken to secure, as far as possible, co-operation and co-ordination between the various hospitals. That wherever possible it is desirable to have paying wards and public Nursing Homes under suitable regulations which shall be open to every member of the profession. That Provident Dispensaries, fully equipped and organised, shall gradually supersede out-patient Departments. That it is desirable to encourage and secure inter-communication and co-operation between all institutions affording medical relief. In brief, that the true principle of admission to a voluntary hospital is, free admission; that each shall be conducted on business principles, under a system, which will render it practically impossible for the funds to be wasted, by providing free relief for any but those who can and do prove their claim, to be entitled to receive it.

### WHAT HAS CREATED THE PRESENT POSITION?

Amongst the *Causes* which have led to the present position of affairs are:—

1. That whereas thirty years ago the mortality in hospitals was very high, and the conditions left much to be desired from the patient's point of view, to-day hospitals are so efficient and hygienically perfect as to make them highly desirable as places of residence for persons suffering from a serious illness. Formerly it was the poverty of the patient, not his will, which brought the majority to the hospitals; to-day matters are exactly reversed.

2. The increased cost of treatment in illness, owing to improved methods and the advances in surgery especially, force many people to seek relief at a free hospital, who would prefer to pay according to their means for such relief in pay wards or public nursing homes, which have become essential for many classes in present circumstances.

3. The facilities for obtaining free hospital treatment are greater and easier to-day than ever before.

4. The competition of hospitals, especially in London in the past, has created a desire to show an increasing number of patients admitted to treatment each year for financial reasons.

5. The visiting medical staffs and teachers at the medical schools have not been actively wishful to reduce the numbers, for educational reasons, which I am advised do not now prevail in regard to medical out-patients at any rate.

6. The attitude of the great body of general practitioners to provident dispensaries has not tended to make these institutions as successful as they might and ought to be.

7. *Finally*, in the absence of an adequate and up-to-date system of provident dispensaries in the populous districts of large cities, patients have acquired the habit of accepting free medical relief at the out-patient departments of the Voluntary Hospitals.

### THE SOLUTION OF THE PROBLEM.

The *Remedy* is to be found in an organisation which will gradually knit together all the existing agencies. The hospitals at present in their out-patient departments give too much or too little relief, from a public point of view. Too much, because many of the patients should be deemed to be ineligible, for the reasons I have already indicated; too little, in the sense that very many of the patients require, and should receive, attention in their own homes through co-operation with a system of provident dispensaries and district nursing associations, including departments for the supply of dressings and appliances at co-operative prices.

The present Conference is full of hope for the future, for it is in my judgment but the beginning of an organised effort, on the part of all the interests concerned, to find and enforce an improved system of medical relief, which shall minimise the misuse of hospitals, make it easy for the really deserving to secure with readiness the medical treatment they are entitled to, and enable all classes of patients to obtain the assistance which their circumstances necessitate. All this can be secured upon a plan, which will encourage thrift and self-reliance, whilst it secures for the patients a guarantee that they will be able to obtain, with readiness, every requisite in the day of sickness. I look forward to the time when the hospital out-patient department will be used mainly for consultative purposes; when we shall have throughout our large towns, and especially in London, an adequate system of up-to-date and efficiently equipped provident dispensaries and district nursing centres, combined, possibly, with a system of insurance, by which all who care to avail themselves of it may readily provide, at a cost within their means, against the day of sickness and accident. I beg to move the resolution.

### THE GENERAL PRACTITIONER'S VIEW.

Dr. Frank M. Pope, F.R.C.P., Chairman of the Hospitals Committee of the British Medical Association, seconded the resolution. He said that Sir Henry Burdett had, from the stores of his knowledge, presented a striking picture of the extent to which free hospital relief was now given; and traced the evils which this naturally entailed on the public generally, the giving as well as the receiving public. In seconding the resolution he wished to address himself to another aspect of the question—namely, the effect of hospital abuse on the status and emoluments of the medical practitioner of this country, and incidentally on the progress and future of medical knowledge in the same area. The unchecked distribution of free medical relief to all and sundry had had a twofold effect on the general practitioner. In the first place, it actually took away his patient; and in the second, it created in the mind of the public an idea, that medical advice was not a thing to be paid for, or to be paid for in such small sums as indicated its comparative worthlessness To an audience such as that he need hardly give instances of the former effects. The cry had gone up, and was still going up with ever-increasing volume, from the general practitioners, that they, though by universal consent better educated and better trained than formerly, found themselves hard put to it to make a living. They saw their contract patients—that was the members of friendly societies—leaving their clubs and going to the out-patient department of the hospitals. They saw their private patients, when a trivial operation was needed that the general practitioner was perfectly competent to perform, and had performed many times during his house surgeoncy period, going off to the in-patient department. Accidents had long ago all drifted to the casualty departments. For instance, the policeman in a street accident never took the person to a medical practitioner, but to the hospital. The deprivation of this surgical practice created a vicious circle, for the general practitioner often became rusty in his surgery, and that, though not more disastrous eventually to the value of his work than becoming rusty in his medicine, was more immediately apparent to his patients; and a fresh argument was furnished to the astute working man who knew a good job when he saw one, and declared again that the hospital was the one and only place where surgical treatment could be effectually carried out. He (the speaker) did not overlook the fact that in many instances the general practitioner contributed to this state of things by himself sending cases to the hospital which might be treated at home—(laughter and hear, hear)—but in many cases this arose from a feeling of hopelessness and of the inevitableness of the existing state of affairs. After all the general practitioner was the first line of defence against sickness, disease, and accident, and in the long run they had to rely on him in the first place. It was false economy to let him get rusty or impose conditions which would result in his being drawn from a less capable class of men. As to the second effect of free medical relief, the lowering of the estimation of the value of medical advice and treatment, there were many evidences. The public, who saw the eagerness with which appointments to voluntary hospitals were sought after, the enormous number of free tickets given them for their subscriptions to these institutions, naturally had doubts as to whether they too should remunerate their own medical men, even in the moderate way they did now. This state of things could not fail to react injuriously on the quality of the work of the backbone of the medical profession, and, if continued, would lead to the universal deterioration of medical work in the country, and a slackening of the proper advance of the medical sciences. The British Medical Association had attempted to deal with this state of things, not in a narrow and selfish spirit, but from the public side as well as the professional. The Association had peculiar advantages for acquiring a knowledge of the actual state of affairs with a view to remedying them. It had branches and correspondents everywhere, and could get valuable information from all hospitals, and it had come to the conclusion, that prompt and resolute action was necessary. The result of the deliberation of the Joint Committee of the first Conference had been before them all in other places, and they were now asked to put the seal of the affirmation of this Conference to the proposals made. They were very moderate, but the British Medical Association did not propose to stop there. There were several other propositions which, though the Joint Committee were not able to accept them at once, they hoped to be able to convert them to in the future, notably the conversion of free dispensaries into provident associations, and the working of these and the existing provident dispensaries as in some way appendages to hospitals; and also the total abolition of small payments at voluntary hospitals as tending to make patients believe they were giving a full *quid pro quo* in relation to their treatment. As to the suitability of patients, he thought that inability to pay for adequate treatment, as stated in the first paragraph of the committee's report, contained the germ of the whole thing. Subscribers' letters were, from the point of view of the British Medical Association, pernicious. They gave the applicant a sense of being treated as of right and not of favour, and made it difficult for the medical staff to give the more deserving a preference over the less. They served eleemosynary purposes, and were given as rewards for Church attendance, and membership of trade unions and shop associations; and when a body of working men received, in return for a subscription, a certain and stipulated number of subscribers' letters, no more and no less for each guinea given, it was impossible to argue—(A voice: "No, no!")—or at any rate to convince them, that they had not a right to exact in return so much attendance. That was his opinion, and he reiterated it. The free gift of the doctor's time was passed over as of no importance. (Hear, hear.) Investigation as to means was, he was glad to say, on the increase, though it was hampered by the existence of letters. The committee had endeavoured to define when relief should cease in cases where any inquiry as to suitability was, in the first instance, impossible. Clause 9, "That where possible the number of new cases to be seen on any one day by an honorary medical officer shall be limited," was an important one. It was very difficult, however, to carry out, but there was no doubt that in many hospitals, from mere pressure of numbers, it was impossible to give careful consideration to each case. Clauses 12 to 19 contained, so far as the committee could see, the germ of a remedy for hospital abuse. The recognition of a hospital as a place where only cases that could not from their nature be treated elsewhere was a necessary antecedent to any remedy. The out-patient department should be fed by provident dispensaries, and subsequent treatment and medicine should be provided by the dispensaries, and not by the hospitals. It would be found desirable that a medical officer of the hospital should attend at the provident dispensary to approve of, and even to select, cases suitable for hospital treatment. The provision of self-supporting nursing homes was perhaps at present in the nature of a pious aspiration rather than a sure and certain hope.

### THE DISCUSSION.

Mr. H. A. Harben, chairman of St. Mary's Hospital, recalled the fact that two years ago a conference of metropolitan hospitals was held to consider means for bringing about better co-operation between the hospitals and provident dispensaries; and they drew up a series of resolutions which in the main agreed with the first clause of the Joint Hospitals Committee's report. Some difficulty, however, was experienced in getting the governing bodies to agree to those proposals, owing to the want of sufficient dispensaries in London and the necessity of careful investigation into those which did exist. He thought that a good many of those present were placed in some difficulty by the form of the resolution proposed. (Hear, hear.) They were asked to express general approval of the principles contained in the proposal; but they were in some difficulty as to extracting the principles from the proposals, and also as to how far they would commit themselves by approval of the priciples to the details of the proposals, to several of which some among them could not agree. For instance, in regard to Clause 13 he thought if they merely said that, subject to proper safeguards, it was desirable to establish pay wards in hospitals, he should be inclined to agree to it. He should divide the proposals into two classes: those connected with hospital abuse in so far as it resulted from indiscriminate charity, and those connected with the relations between medical practitioners and their patients. With regard to the latter, many of those present did not feel called upon to express an opinion without having had the question properly threshed out. (Hear, hear.)

After some discussion it was decided to alter the resolution by substituting for "approves generally" the words "welcomes the further consideration."

Dr. G. A. Heron, of the City of London Hospital for Diseases of the Chest, said Sir Henry Burdett, in his very able speech, had referred to the densely crowded out-patient departments of their hospitals. Everyone connected with hospitals was familiar with this. An out-patient department was a sight worth seeing, though a very sad sight. What could they expect from a house surgeon or physician on duty, say, from two to five, six, or seven o'clock, seeing a constant stream of patients all the time? What man, however gifted, could keep on like that? God help the patient who went last to him! (Hear, hear.) That was a state of things which ought to be remedied, and they need not wait for the report of the committee to do it. They could do it now. They could make it easy to diminish the crowds by taking care that only cases which ought to be seen there were seen. Let them take the case of the chronic dyspeptic. If the medical officer would say to the patient that he could not possibly be treated with success in the out-patient department, that he must either be treated at home or in an in-patient ward—if they had the courage to say this they would at once eliminate an immense number. Take another class: the treatment of consumptives in all stages of the disease in an out-patient department was simply a farce; it could not be done; it was unfair to the doctor, to the patient, and to the healthy community. (Hear, hear.) If they eliminated those people, they got rid of a great deal of the pressure on the out-patient departments. He did not say that they ought never to go, but he did say they ought never to be treated there. After the first examination they should be told, "You must not come back, we have done all we can for you here." If their medical officers were authorised to take up that position they would in ten days get rid of a great deal of the overcrowding, with advantage both to the patients and to the healthy community. (Hear, hear.) Sir Henry Burdett had referred to assurance against sickness and very gently hinted at its necessity; but he (the speaker) was convinced that the strongest possible pressure should be brought to bear on the wage-earner to compel him to assure against sickness, even though it meant going less often to the public-house. (Hear, hear.) He thought that must commend itself to everybody; it was done in other countries with magnificent results. They should ask the Government to bring it still more within the reach of the working man to assure against sickness, accident, and old age. (Cheers.) That would help more than anything else to get over this question of free medical advice. It ought to be the right of every man to be medically attended to when ill, and it ought also to be the right of every medical man to be paid for that attendance. (Hear, hear.) That would put the relationship between doctor and patient on fair and honourable terms. (Hear, hear.) He felt sure that the adoption of these two suggestions would do more to mitigate hospital misuse than anything else.

Dr. H. G. Lys, Bournemouth, said he could not agree to the abolition of subscribers' letters. Under the system adopted at his hospital they had certificates so worded as to lead the governors who signed them to inquire into the circumstances of the applicants and to certify in definite words that they were people entitled to relief. These were distributed in unlimited numbers, and they did not find the system open to abuse. He did not consider it was either desirable or practicable to altogether abolish subscribers' letters. Obviously, the circumstances of the smaller provincial hospitals were different from those in large centres. But he did wish they could find a means to exclude a large number of trivial cases; that would result in great gain to all.

The resolution as altered was then carried unanimously. It was also resolved to hold the conference annually, and after the proposal had been modified by the adoption of several suggestions a Standing Joint Committee of fifteen was appointed to act in conjunction with the Hospitals Committee of the British Medical Association, with instructions to communicate to all hospitals the results of the conference, to arrange for the holding of the conference in 1907, and in the interval to carefully consider the suggestions laid before the meeting with the reports received from the various hospitals. The following were elected to the committee :—

Mr. Walter Baily, University College Hospital; Sir Henry Burdett, K.C.B.; Mr. W. G. Carnt, Derbyshire Royal Infirmary; Mr. Neville Chamberlain, Birmingham General Hospital; Mr. A. W. Davis, Secretary, Hospital Saturday Fund; Mr. Dinwiddie, Dumfries and Galloway Royal Infirmary; Sir Charles Gilman, Norfolk and Norwich Hospital; the Hon. Sydney Holland, London Hospital; Lord Kilmorey, Charing Cross Hospital; Sir Riley Lord, The Infirmary, Newcastle-on-Tyne; Mr. Charles Lupton, General Infirmary, Leeds; Mr. Manfield, M.P., Northampton General Hospital; Mr. Herbert W. Mason, East Suffolk and Ipswich Hospital; Colonel Montefiore, Charity Organisaton Socety; and Mr. Arthur Napper, Cranleigh Village Hospital.

It was also resolved to appeal to hospitals to contribute toward the expenses of carrying out the work.

Thanks to the authorities of University College and to the chairman brought the proceedings to a close.

